# A Few Ideas on Mechanical Dentistry

**Published:** 1859-07

**Authors:** F. J. S. Gorgas


### ARTICLE IV.

### *A Few Ideas on Mechanical Dentistry.* By F. J. S. GORGAS, D. D. S.

SEVERAL articles that have appeared in the Journal of late, show, most conclusively, that practice as well as theory is absolutely necessary to attain to a high degree of skill in our profession.

Dividing our practice into operative and mechanical dentistry, we sometimes find practitioners, who, though they excel in the former branch altogether fail in the latter.

The cause of this with some is, that they attach but little importance to mechanical dentistry, when compared with operating.

This is a grave error, for if we fail to give satisfaction in one branch of what we profess to practice, our reputation suffers to the same degree almost as if we failed in both.

Where our patients are the judges, apart from the observation and opinion of the dentist, I hesitate not in saying that success in the mechanical branch assists the practitioner more in regard to reputation than the same degree of success in operative dentistry.

A filling may be inserted, and provided that filling preserves the tooth, it is in the majority of cases if not entirely forgotten, never spoken of to others, while an artificial denture, giving perfect satisfaction, will not only be at once observed by those already aware of the loss of the natural organs, but be frequently spoken of in the highest terms by the grateful patient.

I do not wish to detract from the praise which skill in either branch so justly merits, but merely to give mechanical dentistry its proper station. Mere mechanical skill, although highly necessary, is not all that is required to perfect one in this branch.

It requires not only an "educated hand," but an "educated eye," and good judgment. Where can we find a nicer task, or one that requires more judgment, than in restoring to the human face its former beauty and natural expression? This is beyond the reach of many, notwithstanding the amount of energy they expend to obtain it.

A knowledge of anatomy and physiology is necessary, which we rarely, if ever, find in the self-made dentist, showing how useful the complete dental education as taught in our colleges, is to him who would rise above mediocrity.

A knowledge of chemistry is also very necessary in our laboratory. I have known practitioners send gold to depots more than one hundred miles distant, to have certain alloys removed, which, owing to their ignorance of chemistry, they were unable to work. Of all professions, dentistry requires the exercise of that great virtue, "patience," and the want of it is the cause of failing with some who are otherwise well qualified. The time spent in giving work a superior finish is not lost even in the most busy season.

As there is much complaint concerning the springing of plates, I wish to say a few words explaining my method of backing and soldering a set of teeth, not claiming it as something new, for I am well aware that many manipulate in the same manner, but merely to explain a method which if followed out will give success.

I first back or line my tooth with platina, rolled as thin as common letter paper, giving the backing the shape I wish it to have when finished, and turning up the base, which is left long for the purpose, about the sixteenth part of an inch, or as wide as I wish the backing to be thick at the base where it joins the plate.

After this is done, I place the tooth in position, and with a burnisher press down upon the plate the part of the platina backing that I have turned up, so as to obtain a perfect fit. Then I bend with plyers the points of the rivets together. I now insert the tooth in equal parts of plaster and asbestos; dry it slowly, and after it is well heated up, melt upon the platina backing by means of the mouth blow-pipe, any scraps of gold I may have after trimming my plate in swaging up. This gives a backing of the same quality as the plate and lessens the amount of solder necessary in the common method, as there are no rivets to solder.

We also have a backing tapering from the grinding surface of the tooth to the base, to which any shape can be given, and which adheres so firmly that the tooth must be broken in small pieces to detach it. If care is taken in heating up and allowing it to cool gradually, no accident is liable to happen. In soldering, I use a copper cup shaped like an impression cup, in the sides and bottom of which are a number of small holes. This cup should be large enough to allow the investment (equal parts of plaster and asbestos in quantity, the asbestos ground fine in a mortar) to be from one-quarter to one-half an inch in thickness around the teeth, aud from three-quarters to one inch deep under the plate. I first fill the cup half full of the investment, mixed rather thick, and taking up the set of teeth fill in the concave surface of the alveolar ridge. I then place the set in cup upon the investment I have already put there, and fill in between the teeth and the side of the cup.

After I have backed my teeth I commence heating up by placing the piece in the oven of a stove, and allow it to remain there three or four hours, after which I use a small charcoal hand furnace, and with a Lawson self-acting blow pipe, heat it up to a high degree, using a mouth blow-pipe to flow the solder.

In heating up with the self-acting blow-pipe, I shift the flame from side to side, by rotating my furnace so as to have an equal degree of heat in all parts of the piece. I allow it to cool before taking it from the furnace.

**NOTE.—The method of backing teeth with platina, as noticed above, was described many years ago in the Journal, by Mr. Wilson, of Edinburg.**

